# Viscosity Estimation of a Suspension with Rigid Spheres in Circular Microchannels Using Particle Tracking Velocimetry

**DOI:** 10.3390/mi10100675

**Published:** 2019-10-04

**Authors:** Misa Kawaguchi, Tomohiro Fukui, Kenichi Funamoto, Miho Tanaka, Mitsuru Tanaka, Shigeru Murata, Suguru Miyauchi, Toshiyuki Hayase

**Affiliations:** 1 Department of Mechanical Engineering, Kyoto Institute of Technology, Kyoto 606-8585, Japan; d9821001@edu.kit.ac.jp (M.K.); m9623015@edu.kit.ac.jp (M.T.); mtanaka@kit.ac.jp (M.T.); murata@kit.ac.jp (S.M.); 2 Institute of Fluid Science, Tohoku University, Sendai 980-8577, Japan; funamoto@tohoku.ac.jp (K.F.); miyauchi@reynolds.ifs.tohoku.ac.jp (S.M.); hayase@ifs.tohoku.ac.jp (T.H.)

**Keywords:** suspension, rheology, power-law fluid, circular microchannel, pressure-driven flow, particle tracking velocimetry, microstructure

## Abstract

Suspension flows are ubiquitous in industry and nature. Therefore, it is important to understand the rheological properties of a suspension. The key to understanding the mechanism of suspension rheology is considering changes in its microstructure. It is difficult to evaluate the influence of change in the microstructure on the rheological properties affected by the macroscopic flow field for non-colloidal particles. In this study, we propose a new method to evaluate the changes in both the microstructure and rheological properties of a suspension using particle tracking velocimetry (PTV) and a power-law fluid model. Dilute suspension (0.38%) flows with fluorescent particles in a microchannel with a circular cross section were measured under low Reynolds number conditions (*Re* ≈ 10^−4^). Furthermore, the distribution of suspended particles in the radial direction was obtained from the measured images. Based on the power-law index and dependence of relative viscosity on the shear rate, we observed that the non-Newtonian properties of the suspension showed shear-thinning. This method will be useful in revealing the relationship between microstructural changes in a suspension and its rheology.

## 1. Introduction

The rheological properties of a suspension (solid particles dispersed in a fluid) vary depending on the particle volume fraction, particle shape, interactions between particles, spatial arrangement of particles, and nature of the solvent [1]. Non-Newtonian phenomenon is commonly accompanied by shear-thinning [2] or shear-thickening [3] behavior, or yield stress [4] resulting from the complex influence of many factors. Suspension flows are ubiquitous in industry and nature. In the industrial field, the rheology of suspension directly influences product quality such as food and paint [5]. Furthermore, suspensions are also important for industrial processes such as filtration [6] and ceramic processing [7,8], since they are the precursors to the manufacturing of numerous products. In nature, examples of suspensions include mud, which is essentially a dispersion of rigid particles, and blood, which is a suspension of red blood cells dispersed in plasma [9,10]. Therefore, understanding rheological properties of suspensions is expected to lead to control of suspension rheology and elucidation of hemorheology.

When the size of particles increases with respect to the channel size, wall effects and interactions between particles are observed. Sangani et al. [11] reported that the presence of walls increases the particle stresslet. Fukui et al. [12] found that particles rotate to achieve a kinetic balance with the surrounding hydrodynamic forces, which results in a decrease in fluid resistance. Moreover, some researchers reported that viscosity significantly decreased for strongly confined conditions [13,14,15,16]. The above phenomena are reflected in the strong correlation between suspension viscosity and microstructure defined by spatial particle arrangements.

Many researchers have addressed the connection between the microstructure and rheology of a suspension [1,17,18]. A numerical approach is useful in assessing the relationship between microstructure and rheology. It is easy to obtain information on the microstructure of a suspension because particle distribution can be captured accurately at all times. Fukui et al. [19] conducted a two-way coupling simulation and reported that non-Newtonian properties change because of particle migration. However, the experimental evaluation of the microstructure is difficult. Light scattering techniques are often used to obtain the structure factor of suspensions, and, although these techniques are useful for colloidal suspensions, their application to non-colloidal suspensions becomes difficult because the size of the particles is large compared with the wavelength of light [20]. Talini et al. [21] proposed a nuclear magnetic resonance (NMR) technique for non-colloidal suspensions, which is capable of measuring structures up to 1 mm. However, this technique could only be applied to static conditions because particles must stay in their positions during the scanning. Thus, for non-colloidal suspensions, it is difficult to evaluate the influence of the change of the microstructure on the rheological properties affected by the macroscopic flow field.

Various methods for measuring viscosity in complex fluids using microfluidic devices have been proposed [22], and several studies have evaluated the velocity field of complex fluids using particle imaging velocimetry (PIV) [23,24,25]. Degré et al. [24] evaluated the viscosity of polymer solutions by PIV. Jesinghausen et al. [26] report that the viscosity evaluated using PIV was 10%–20% lower than the value measured by a viscometer. These studies were conducted using rectangular channels. However, from an engineering and hemorheological point of view, it is important to consider the relationship between particle distribution and rheology in circular channels of pipe flow.

The purpose of this study is to propose a new method for evaluating both the microscopic structure and viscosity of non-colloidal hard sphere suspension flows. In this paper, a microchannel with a circular cross section was fabricated, and the suspension flow with fluorescent particles was measured using a microscope. The velocity distribution was obtained using a particle tracking velocimetry (PTV), and the particle radial distributions were also determined. The non-Newtonian properties of a suspension were examined using the power-law fluid model.

## 2. Materials and Methods

### 2.1. Suspension

Dilute suspensions of rigid spherical particles in a Newtonian fluid were used. Fluorescent polymer microspheres with a diameter *d*_p_ = 25 μm (35-5, Thermo Fisher Scientific, Waltham, MA, USA) were suspended in a mixture of water and glycerol (075-00616, Wako, Osaka, Japan) to attain neutral buoyancy. The density of the mixture was equivalent to that of the particle density (1.05 g/cm^3^). A small amount (<0.5%) of surfactant Tween 20 (17-1316-01, GE Healthcare, Chicago, IL, USA) was added to fully disperse the dry powder of fluorescent particles. The volume fraction (*ϕ*) of the suspended particles measured by using a chip (C-Chip, NanoEntech, Seoul, Korea) was 0.38%. The suspension viscosity was 1.73 mPa·s, which was measured using an oscillation-type viscometer (VM-10A, CBC Co., Ltd., Tokyo, Japan) at room temperature (27 °C).

### 2.2. Microchannel

**Figure 1:**
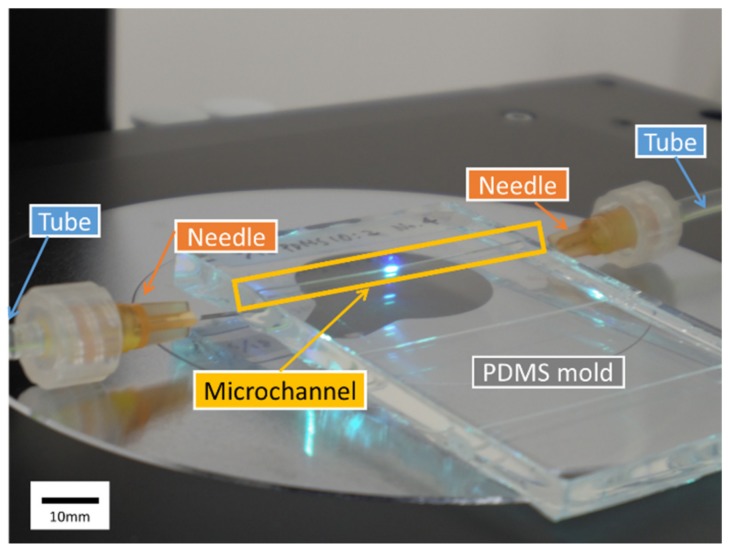
Circular polydimethylsiloxane (PDMS) microchannel in the PDMS mold. Needles were inserted on both sides of the microchannel and connected by tubes.

Circular cross section polydimethylsiloxane (PDMS) microchannels (Figure 1) were fabricated using a fishing gut [27]. Most PDMS microfluidic channels are fabricated using photolithographic techniques and their cross section is typically rectangular [28]. Recently, some methods [29,30] have been developed to form more complex microchannels. Because pipe flow is important for many applications, such as industrial or blood flow, circular cross section PDMS microchannels are often used [31,32,33]. The fabrication of a circular PDMS microchannel using a fishing gut is explained below.

(1) Holes were drilled in a case (Styrol square case type 3, As One, Osaka, Japan) and a fishing gut with a diameter *D* = 520 μm (Type 10, Matsuura Industry, Osaka, Japan) was passed through the holes.
(2) PDMS (Silgard 184 Silicone Elastomer Kit, Dow Corning, Midland, MI, USA) was synthesized by mixing the elastomer base with its curing agent in a weight ratio of 10:2. The ratio of the curing agent was increased to twice the recommended value to suppress the effects of PDMS elasticity. Next, PDMS was poured into the case and cured in an oven (75 °C) overnight.
(3) Lastly, the gut was pulled out gently and the PDMS mold with the microchannel was taken out from the case. For the measurement, needles (NN-2516R, TERUMO, Tokyo, Japan) were inserted on both sides of the microchannel and connected by tubes.

### 2.3. Experimental Setup

**Figure 2:**
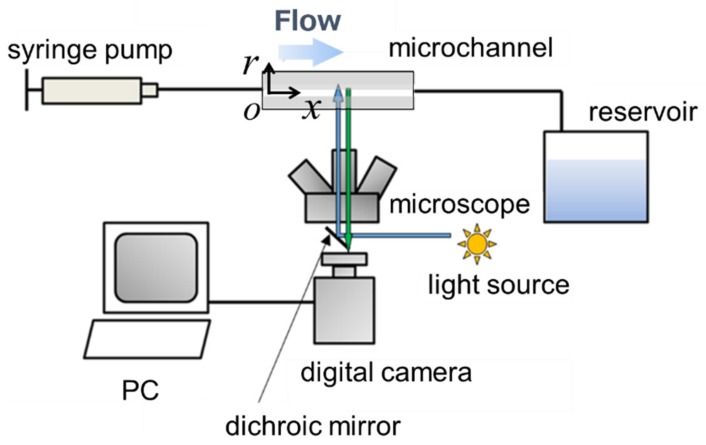
Schematic view of experimental apparatus. The measurement area was set parallel to the flow direction passing through the tube axis to measure both particle distribution in the radial direction and flow velocity.

Figure 2 shows a schematic of the experimental apparatus. A suspension was injected into the microchannel by a syringe pump (KDS210, KD Scientific, Holliston, MA, USA) in a steady state. Fluorescent images of the flow field and phase contrast images were obtained for calibration. The Reynolds number was altered by presetting the flow rate to approximately 5–20 µL/min, which corresponds to the *Re* range from 0.125 to 0.5. In this study, confinement *C* [11] is defined as *C* = *d*_p_*/D* = 0.05, which is the ratio of the particle size to tube size. The particle Reynolds number (*Re*_p_) [34] is defined as *Re*_p_ = *Re* × *C*^2^ and it corresponds to *Re*_p_ in the range of 2.9 × 10^−4^–1.2 × 10^−3^.

The measurement area was set parallel to the flow direction passing through the tube axis. The particle distribution in the radial direction and flow velocity were measured. To eliminate the influence of the attachment of the connector on the tube, the flow field was measured at a location 20*D* away from the inlet. Measurement conditions were fixed as recorded in Table 1. Different frame rates were set for each condition so that the particles travel similar distances (about 0.3*D* on the centerline) at each shutter interval. Furthermore, the flow fields were measured three times for each condition using a disk scanning microscope system (IX83-DSU, Olympus, Tokyo, Japan) at 10× magnification.

### 2.4. Image Processing

**Figure 3:**
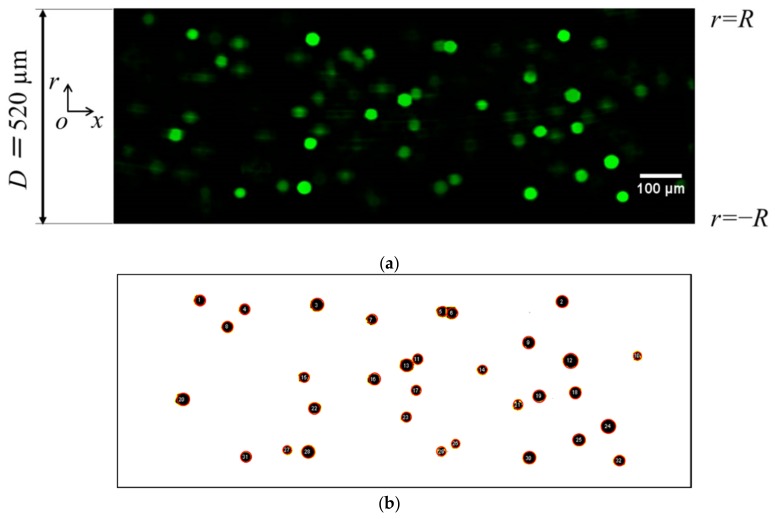
Representative images of particles in a microchannel. (**a**) Fluorescent image after background subtraction and (**b**) a sample of extracted particles in a binary image. Particles are encircled in red in Figure 3b for clarity.

Figure 3 shows the sample of images used for analysis. The procedure for obtaining particle concentration profiles and axial velocity profiles using image processing software (ImageJ/Fiji, National Institutes of Health (NIH), Bethesda, MD, USA) [35,36] is as follows.

1. Fluorescent particle images were converted to binary images using the “subtract background” command given as a preparation, and the binarization was conducted based on the threshold determined using the Otsu method [37].
2. Particle size for analysis was set to 200 pixel^2^, which corresponds to the particle diameter of ~ 22 µm, and the coordinates of the particle center point were extracted using macros. Figure 3b shows a sample of extracted particles obtained from a binary image. The particles are encircled in red in Figure 3b for clarity. In this step, “watershed segmentation” was used for identifying each particle, and the function of automatically separated or cut apart particles were recognized as a single cluster due to overlapping.
3. The measurement region was equally divided in the radial direction, and the number of particles in each section was counted from their radial positions. The existence probability of particles in the radial direction *σ* was then obtained with respect to the number of particles in the entire image.
4. Steps 1–3 were repeated for all images, and these data were analyzed for a time-averaged particle concentration profile.
5. PTV was also employed, and time-averaged velocity profiles were obtained. The non-Newtonian properties of the suspension were evaluated by comparing the time-averaged measured velocity profiles with those from a power-law fluid. Details have been provided in Section 2.5.

Sizes of the particles in the images were certainly different from each other, as shown in Figure 3. This could be attributed to the particle distance with respect to the focus plane of the microscope. Since the apparently large particles might be out of the focal area, the translational velocity of the particles could be decreased when compared to the theoretical value. On the other hand, the apparent particle size variation was also caused by the difference in the refractive index between the fluid and the microchannel. The refractive index of PDMS is 1.41 while that of water is 1.33, or 1.47 for glycerol. The refractive index of a water/glycerol mixture of 61% glycerol matches that of PDMS [30,38]. However, in order to satisfy the neutrally buoyant condition according to priority, a water/glycerol mixture with approximately 20% glycerol was used in this study. Therefore, the refractive index of the fluid may not be well-matched with that of the microchannel. Nevertheless, the error of the flow rate was, at most, 5%, as described later, and these effects might be negligible in this study.

### 2.5. Non-Newtonian Properties

A power-law model is a simple model used to represent viscosity as a function of the shear rate. The non-Newtonian properties of the suspension were assessed by comparing the measured velocity profiles acquired by PTV with those from a power-law fluid. The velocity profiles of power-law fluids [39] are as follows.

(1) $$u\left(r\right)=\frac{3n+1}{n+1}u_{0}\left[1-{\left|\frac{r}{R}\right|}^{\frac{n+1}{n}}\right],$$

where *u*(*r*) is the axial velocity, *n* is the power-law fluid index, *u*_0_ is the characteristic velocity, *R* is the radius of the microchannel, and *r* is the radial position in a range satisfying the condition *– R* < *r* < *R*. If *n* = 1, the fluid is Newtonian. Otherwise, it is classified as a non-Newtonian fluid, which exhibits shear-thinning (thixotropy) for *n* < 1 and shear-thickening (dilatancy) for *n* > 1.

Power-law fitting was applied using the least-absolute-value method [12] to minimize the following cost function.

(2) $$\mathrm{cost}\;\mathrm{function}\;=\;\sum_{\mathrm{all particles}}\left|u_{m}\left(r\right)-u\left(r\right)\right|\to \min.,$$

where *u*_m_(*r*) is the measured axial velocity and *u*(*r*) is the axial velocity estimated using the power-law fluid equation (Equation (1)) at the corresponding radial position. The cost function was defined as a sum of the absolute differences for all particles.

### 2.6. Relative Viscosity

The relative viscosity was assessed using the viscosity equation for power-law fluids. The effective viscosity *η*_eff_ is defined in the following equation.

(3) $$\eta_{\mathrm{eff}}=\eta_{0}{\dot{\left|\gamma \right|}}^{n-1},$$

where *η*_0_ is the viscosity of a suspension, and $\dot{\gamma }$ is the shear rate. Note that when $\dot{\gamma }$ = 1, the relative viscosity equals 1, regardless of the power-law fluid index *n*.

The viscous resistance is equivalent to the pressure drop in a fully developed laminar tube flow. For dilute suspensions, the drag force acting on the particles can be neglected when the suspension flows steadily [12], and the flow energy dissipates on the walls. Thus, the pressure drop is equal to the spatially integrated wall shear stress value. The wall shear stress can be calculated from the shear rate on the walls using Newton’s law, and, therefore, the relative viscosity *η*_eff_/*η*_0_ can be approximated using the shear rate on the wall. The shear rate on the wall is analytically expressed in Equation (4), and the relative viscosity *η*_eff_/*η*_0_ was evaluated from the equation using the power-law index *n* by the velocity profile fitting.

(4) $$\frac{\eta_{\mathrm{eff}}}{\eta_{0}}={\left.{\left|\frac{\partial u}{\partial r}\right|}^{n-1}\right|}_{r=-R}={\left(\frac{3n+1}{n}\frac{u_{0}}{R}\right)}^{n-1}.$$

## 3. Results and Discussion

**Figure 4:**
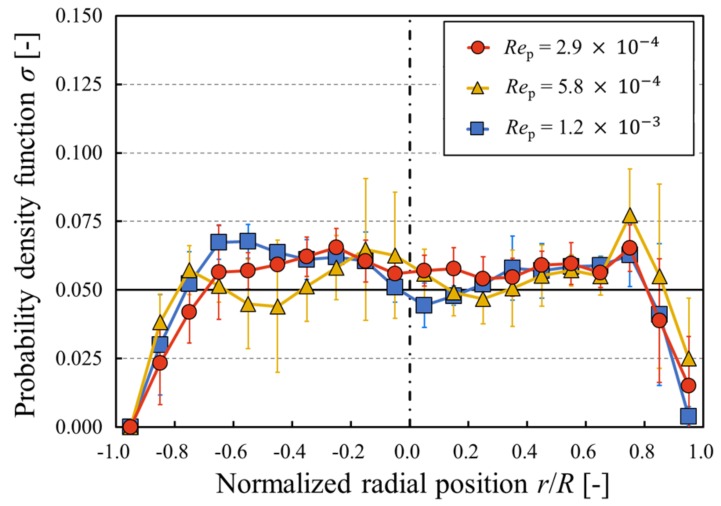
Concentration profiles. The data plotted are the mean ± 1 SD. The solid black line represents uniform distribution, and the black dash–dot line at *r*/*R* = 0.0 indicates the center of the microchannel.

Figure 4 shows the particle concentration profiles in the radial direction for different particle Reynolds number conditions. In this study, the number of divisions in the radial direction was set to 20, which is approximately equivalent to the ratio of the channel diameter to the particle size (=1/*C*). Therefore, the value of the probability density function becomes *σ* = 1/20 = 0.05 (shown with a solid black line) when the spatial arrangement of suspended particles is uniform. Figure 4 shows that the particles were distributed around *σ* = 0.05 with minor deviation, regardless of the particle Reynolds number.

Choi et al. [40] investigated the distributions of neutrally buoyant spherical particles suspended in a micro-scale pipe flow using a digital holography technique, which showed a qualitatively similar distribution when compared to our experimental results.

The following reduced tube length *L*_3_ is defined by Segré and Silberberg [41].

(5) $$L_{3}=Re\left(\frac{L}{D}\right){\left(\frac{d_{p}}{D}\right)}^{3},$$

where *L* is the distance from the inlet of a microchannel. Choi et al. [40] observed that the particle distribution changes due to an inertial effect under the following condition.

(6) $$L_{3}\geq 3.$$

In this study, the reduced tube length *L*_3_ was in the order of 10^−4^–10^−3^, and, hence, slight changes in radial particle distribution can be observed due to weak inertial effects. Fukui et al. [42] in their numerical study demonstrated that the dispersion of the particles was relatively uniform under low particle Reynolds number conditions. In this study, a mixture of water and glycerol was selected as a solvent for neutrally buoyant conditions. A similar distribution has been confirmed for low concentration suspensions under neutrally buoyant conditions by Yan and Koplik [43].

**Figure 5:**
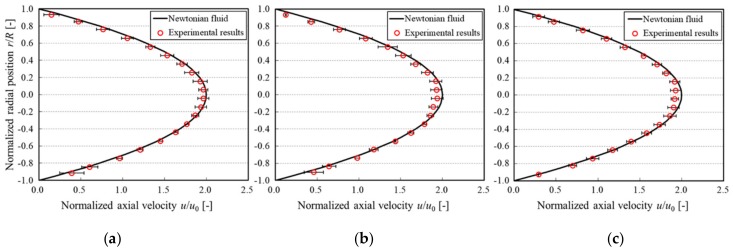
Normalized axial velocity profiles. The red plots and error bars are the mean ± 1 SD, and the black solid line is the velocity profile of Newtonian fluid (*n* = 1) for comparison. (**a**) *Re*_p_ = 2.9 × 10^−4^, (**b**) *Re*_p_ = 5.8 × 10^−4^, and (**c**) *Re*_p_ = 1.2 × 10^−3^.

Figure 5 shows the axial velocity profiles for each particle Reynolds number condition. The measured flow rate decreased by, at most, 5% when compared with the presetting flow rate value. When the measured velocity profiles were compared with those from the power-law fluid model, the velocity profile (Equation (1)) with an equivalent measured flow rate was applied. Moreover, the fully developed boundary layer can be observed because the length of the measurement area from the inlet of the tube was sufficiently long when compared with the inlet length.

From Figure 5, it can be seen that the velocity profiles were slightly blunted with an increasing particle Reynolds number. Hampton et al. [44] experimentally observed blunted velocity profiles. Jabeen et al. [45] reported a similar trend by numerical approach. Moreover, it has also been observed that velocity profiles become more blunted at high concentration conditions [43,46]. In this study, the suspension was dilute. Therefore, the velocity profiles were observed to be slightly blunted.

**Figure 6:**
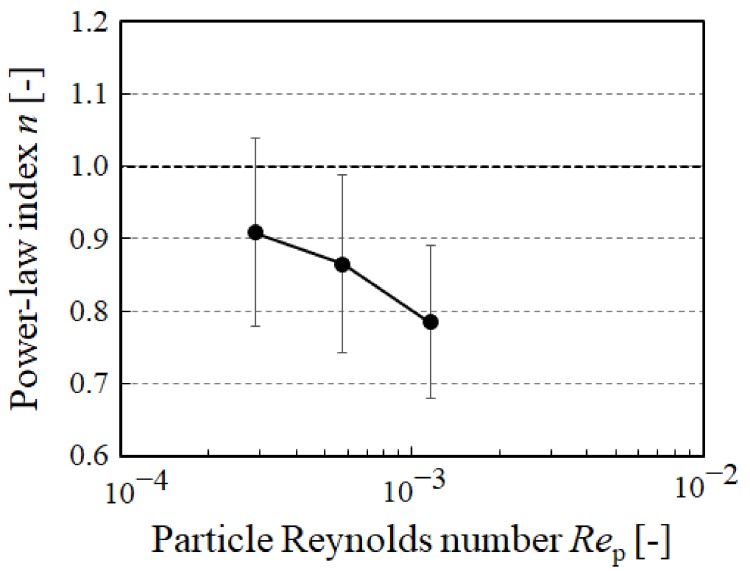
Relationship between power-law index *n* and particle Reynolds number *Re*_p_. The plots are the mean ± 1 SD.

Figure 6 shows the relationship between the power-law index *n* and particle Reynolds number. The power-law fluid index decreases with an increasing particle Reynolds number. Although the experimental conditions were limited, this tendency was consistent with that of the previous study [42] by numerical simulation. Non-Newtonian properties of the suspension showed shear-thinning because the power-law index *n* is smaller than 1. Moreover, it is considered that the shear-thinning is more enhanced with an increasing particle Reynolds number.

**Figure 7:**
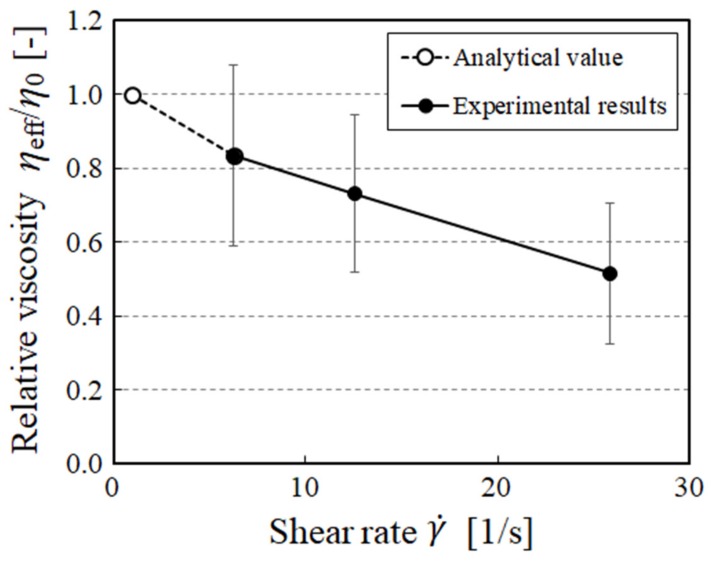
Relationship between relative viscosity *η*_eff_/*η*_0_ and the shear rate $\dot{\gamma }$. The plots are the mean ± 1 SD. The relative viscosity was estimated using Equation (4).

Figure 7 shows the relationship between relative viscosity *η*_eff_/*η*_0_ and shear rate $\dot{\gamma }$. In this study, because non-Newtonian properties were evaluated by the power-law fluid, the relative viscosity equals 1 when $\dot{\gamma }$ = 1 analytically (Equation (3)). This is also plotted in Figure 7 as an analytical value. From Figure 7, it was found that the relative viscosity *η*_eff_/*η*_0_ decreased with an increasing shear rate $\dot{\gamma }$.

The viscosity of the suspension was determined from the velocity distribution using the power-law fluid model. Degré et al. [24] measured the viscosity of polymer solutions using PIV. Jesinghause et al. [26] applied a similar method for suspension flow in a rectangular channel. In this study, the viscosity of the suspension flow in a circular channel was evaluated by PTV. The benefit of using PTV lies in its application to the dilute suspensions or to a suspension with larger particles. In this study, we focus on the relationship between the microstructure and the consequential rheological properties of a suspension. Especially, we also focus on change in microstructure due to inertia. In order to investigate the inertial effects of suspended particles, experimental works should be conducted for a wide range of particle Reynolds number conditions. Particle Reynolds number can be changed by two parameters, i.e., flow velocity or particle size. If larger particles are used, the number of particles are decreased for the same particle concentration condition. When the number of particles contained in an interrogation window is not enough, accuracy of the PIV analysis becomes poor. Therefore, in this sense, PTV may be preferable to PIV. Moreover, one of the advantages of our method is that it is capable of evaluating rheological properties while considering the microstructure changes of the suspension, which is difficult with a classical rheometer. It has been reported in previous research that wall shear stress decreases locally in micro-vessels due to the motion of blood cell and hydrodynamic interactions [47,48]. Furthermore, in numerical simulations, the relative viscosity could be below 1 locally for dense (highly concentrated) suspensions with rigid particles [16]. These findings are important for considering blood flow in bioengineering. Endothelial cells covering the inner surface of blood vessels sense the wall shear stress (WSS) and respond to it mechanically. For example, when the WSS increases, endothelial cells produce vasodilators, such as nitric oxide (NO) [49]. It is also suggested that endothelial cells can sense changes in WSS not only temporally but also spatially [50]. Therefore, it is important to understand the local WSS distribution and the local viscosity changes. On the other hand, this proposed method is limited in the case of non-transparent fluid or highly concentrated suspension.

**Figure 8:**
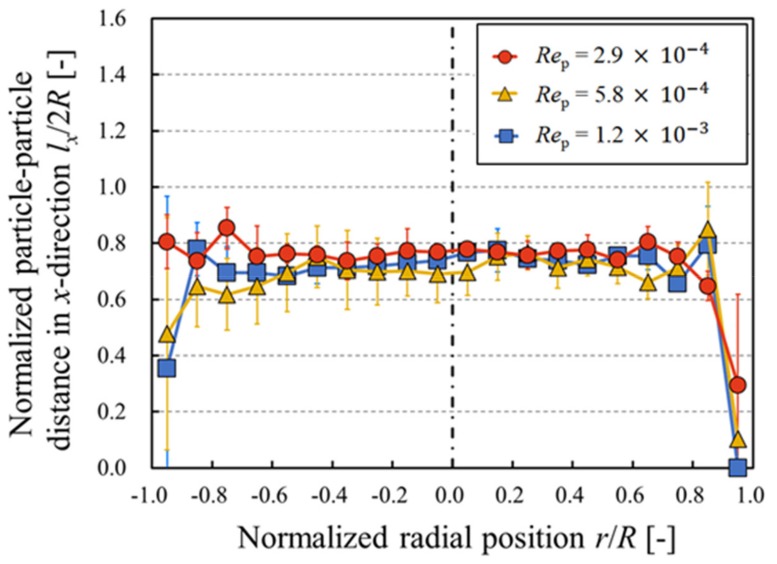
Spatially averaged axial distances between particles referring to the radial direction. The data plotted are the mean ± 1 SD. The value of distance was normalized by the diameter of the microchannel.

Figure 8 shows the spatially averaged axial distances between particles referring to the radial direction. Note that the particle–particle distance was normalized by the channel diameter. The difference in the distance referring to the radial direction was not significant. In addition, these distributions are independent of the particle Reynolds number. Doyeux et al. [13] mentioned that the intrinsic viscosity can be expressed as a function of the particle–wall distance. In order to consider suspension rheology in terms of the microstructure, the radial particle distributions have been investigated in many studies. On the other hand, according to Fukui et al. [12], when the rotational velocity decreases due to the particle–particle interactions, the macroscopic viscosity of the suspension increases. Therefore, it is important to consider particle–particle distance not only in the radial direction but also in the axial direction in order to evaluate the microstructure and consequential rheological properties.

Microstructure is key to understanding the mechanism of suspension rheology [1,17,18]. Red blood cells move to the axis of the blood vessel due to their deformability, which is known as axial accumulation [51]. On the other hand, for rigid particles, when the bulk or particle Reynolds number increase, particles begin migrating due to inertia toward the radial equilibrium positions at 0.6*R*. When particle migration occurs, the distribution of particles in the cross section becomes annulus, which is known as the “tubular pinch effect” or the “Segré–Silberberg effect” [41,52]. Analysis by numerical approaches suggest that the non-Newtonian properties are altered due to these microstructure changes [42,45]. According to previous research, the equilibrium position is around 0.6*R*, and the equilibrium position shifts toward the wall with an increasing Reynolds number [53,54], while it shifts toward the tube axis with increasing confinement [54]. In the future works, we will elucidate the influence of these equilibrium positions, i.e., the difference in microstructure, on the suspension rheology in detail.

In previous studies, velocimetry-based viscometers rely on measuring the velocity profile at a given pressure drop. The shear rate can be calculated from the gradient of the velocity profiles (Equation (4)), and shear stress was calculated from the pressure drop. A curve of shear stress versus the shear rate can be obtained directly. Thus, the previous method allows for direct quantification of the rheology of complex fluids [22]. On the contrary, in the proposed method, the relative viscosity is examined using the power-law fluid model in consideration of the microstructure. However, only a qualitative evaluation of non-Newtonian properties of a suspension is implemented in this paper. Therefore, quantitative validation of our method is required. This includes measuring the relative viscosity using a Couette rheometer or introducing a pressure sensor to the experimental system as seen in previous research [24,26]. Such quantitative examinations will be necessary in future works.

## 4. Conclusions

Suspension flows containing fluorescent particles in a microchannel with a circular cross section were measured. We proposed a new method for evaluating the changes of the microstructure and rheological properties of a suspension using PTV and a power-law fluid model. The distribution of suspended particles in the radial direction was obtained from measured images. In addition, non-Newtonian properties of the suspension were evaluated using the velocity distribution obtained by PTV and the power-law fluid model. This method is useful for revealing the relationship between microstructural changes of a suspension and rheology.

## Figures and Tables

**Table 1:** Measurement conditions.

*Re* (-)	*Re*_p_ (-)	Time (s)	Frame Rate (fps)
0.125	2.9 × 10^−4^	200	5
0.25	5.8 × 10^−4^	100	10
0.5	1.2 × 10^−3^	50	20
